# Supplementary material for: Allelochemical Activity of Eugenol-Derived Coumarins on Lactuca sativa L
Source: Plants (Basel). 2020 Apr 20;9(4):533. doi: 10.3390/plants9040533 (PMC7238165; doi:10.3390/plants9040533)
Supplement: Supplementary file 1 [file plants-09-00533-s001.pdf]

Allelochemical activity of eugenol-derived coumarins on *Lactuca sativa* L.

Supplementary File 1: Synthesis, purification, and characterization data of coumarins A1–A6; and Figure S1: Antioxidant enzyme activity and lipid peroxidation. (a) Superoxide dismutase (SOD) activity in Lactuca sativa seedlings exposed to the different concentrations of coumarin A1. (b) Catalase (CAT) activity in Lactuca sativa seedlings exposed to the different concentrations of coumarin A1. (c) Quantification of lipid peroxidation in seedlings of Lactuca sativa exposed to the different concentrations of coumarin A1. Columns followed by the same letter do not differ statistically by the Scott–Knott test at 5% significance. Bar: standard error.

1. CHEMISTRY

1.1. Generalities

All required chemicals were purchased from Sigma-Aldrich (Sao Paulo, Brazil) and used without further purification. Thin-layer chromatography (TLC) on sílica gel TLC plates (ALUGRAMR Xtra Sil G/UV254, Macherey-Nagel) was used to check purity of the obtained compounds and to monitor the reactions progress. The spots were detected by exposure to the UV light at 254 nm. For column chromatography, column grade (0.040–0.063-mm mesh size) silica gel was employed (Sorbiline R) and the mobile phases are described in each experiment. Melting points of the compounds were obtained on a PFM-II melting point apparatus (MS Tecnopon, Piracicaba, Brazil) and are uncorrected. IR spectra were recorded on a FT-IR- Affinity-1 spectrometer with an ATR dispositive (ShimadzuR, Kyoto, Japan). NMR spectra were recorded on a Bruker AC-300 spectrometer (Rheinstetten, Germany) operating at 300 MHz for ^{1}H-NMR and 75 MHz for ^{13}C-NMR spectra. Chemical shifts are expressed as δ (ppm) relative to TMS as the internal standard. The coupling constants (J) are given in Hertz. Multiplets are given as s (singlet), d (doublet), dd (doublet of doublets), t (triplet), q (quartet), sex (sextet), and m (multiplet).

1.2. Synthesis, purification and characterization data for Coumarins A1-A6

1.2.1. Synthesis of the intermediate formyleugenol

Hexamine (5 eq) was solubilized in glacial acetic acid (40 mL) and this solution was stirred at 125 °C for 10 min, then eugenol (1 eq) was added in one portion. Reaction was maintained at 125 °C for 5 h under stirring. Following, 2 M HCl (10 mL) was added and the reaction mixture was maintained as such for additional 30 min. Then, this mixture was cooled down to 25 °C, washed with aqueous $NaHCO_3$, and extracted with dichloromethane. The organic phase solvente was evaporated in a rotary evaporator and the oil purified by column chromatography (hexanes : ethyl acetate, 9:1, v/v).

Light greenish oil; yield 57%. IR (ATR) 3200–2800 (ν O-H phenol), 3005 (ν C-H ar), 2965 (ν C-H sp^3), 2850 (ν C-H aldehyde), 1655 (ν C=O aldehyde), 1593 (ν C=C ar), 1265 (ν C-O-C). ^{1}H NMR ($CDCl_3$ 300 MHz) δ 10.96 (s; 1H), 9.87 (s; 1H), 6.97 (s; 1H), 6.94 (s; 1H), 5.87–6.01 (m; 2H), 5.07–5.13 (m; 1H), 3.90 (s; 3H), 3.37 (d, $J_{H9,H10}$ 6.6 Hz; 2H). ^{13}C NMR ($CDCl_3$ 75 MHz) δ 196.73, 150.19, 148.34, 136.85, 131.55, 123.83, 120.58, 118.82, 116.66, 56.41, 39.53.

1.2.2. General procedure for the synthesis and purification of coumarins A1-A4

The specific β-ketoester or malonic acid (1 eq.), formyleugenol (1 eq.) and piperidine (0.1 eq) were added to ethanol, mixtured and heated to 80 °C in a glycerol bath until TLC indicated the end of the reaction (hexane : ethyl acetate; 8:2, v/v). The remaining slurry solid was purified by filtration

and trituration with diethyl ether until a fine powder was formed, which was again isolated by simple filtration.

6-(prop-2-en-1-yl)-8-methoxy-2-oxo-2H-chromene-3-carboxylic acid (A1)

From malonic acid. Light yellowish solid; yield 61%. M.p.: 156-157 °C. IR (ATR) 3075-2750 (ν O-H carboxilic), 3046 (ν C-H ar), 3019 (ν C-H sp^2), 1743 (ν C=O ester), 1669 (ν C=O carboxilic). ^{1}H NMR ($CDCl_3$ 300 MHz) δ 8.8336 (s, 1H), 7.1045 (s, 1H), 7.0906 (s, 1H), 3.9857 (s, 3H), 3.4672 (d, 2H, $J_{H13,H14}$ 6.66 Hz), 6.0160-5.8814 (m, 1H), 5.1951-5.1013 (m, 2H). ^{13}C NMR ($CDCl_3$ 75 MHz) δ 163.82, 162.74, 151.72, 147.33, 142.87, 138.83, 135.83, 120.64, 119.01, 117.97, 117.61, 115.12, 56.57, 39.57.

3-acetyl-8-methoxy-6-(prop-2-en-1-yl)-2H-chromen-2-one (A2)

From ethyl acetoacetate. Dark orange solid; yield 24%. M.p.: 172-173 °C. IR (ATR) 3072 (ν C-H ar), 3006 (ν C-H sp^2), 1712 (ν C=O ester), 1678 (ν C=O ketone). ^{1}H NMR ($CDCl_3$ 300 MHz) δ 8.4251 (s, 1H), 7.0070-7.0002 (m, 2H), 3.9600 (s, 3H), 3.4270 (d, 2H, $J_{H13,H14}$ 6.51 Hz), 6.0118-5.8777 (m, 1H), 5.1570-5.0919 (m, 2H), 2.7106 (s, 3H).^{13}C NMR ($CDCl_3$ 75 MHz) δ 195.80, 158.99, 147.84, 147.04, 143.75, 137.25, 136.29, 124.74, 120.71, 118.77, 117.19, 116.82, 56.47, 39.81, 30.72.

3-benzoyl-8-methoxy-6-(prop-2-en-1-yl)-2H-chromen-2-one (A3)

From ethyl benzoylacetate. White solid; yield 83%. M.p.: 168-169 °C. IR (ATR) 3048 (ν C-H ar), 2962 (ν C-H sp^2), 1713 (ν C=O ester), 1659 (ν C=O ketone). ^{1}H NMR ($CDCl_3$ 300 MHz) δ 8.0025 (s, 1H), 7.8739-7.8417 (m, 2H), 7.5914 (tt, 1H, $J_{H4',H3'}$ 7.41 Hz, $J_{H4',H2'}$ 2.61 Hz), 7.4797-7.4292 (m, 2H), 7.0013 (s, 1H), 6.9675 (s, 1H), 6.0253-5.8908 (m, 1H), 5.1600-5.0908 (m, 2H), 3.9684 (s, 3H), 3.4365 (d, 2H, $J_{H13,H14}$ 6.63 Hz). ^{13}C NMR ($CDCl_3$ 75 MHz) δ 191.89, 158.12, 147.19, 145.76, 143.14, 137.25, 136.35, 133.86, 129.70, 128.66, 127.28, 119.80, 118.67, 117.11, 116.14, 56.46, 39.82.

8-methoxy-3-(4-nitrobenzoyl)-6-(prop-2-en-1-yl)-2H-chromen-2-one (A4)

From ethyl 4-nitrobenzoylacetate. Light greenish solid; yield 96%. M.p.: 173-174 °C. IR (ATR) 3074 (ν C-H ar), 3038 (ν C-H sp^2), 1716(ν C=O ester), 1677 (ν C=O ketone), 1522 and 1350 (ν O=N-O). ^{1}H NMR ($CDCl_3$ 300 MHz) δ 8.2870 (d, 2H, $J_{H3',H2'}$ 8.56 Hz), 8.2169 (s, 1H), 7.9628 (d, 2H, $J_{H2',H3'}$ 8.56 Hz), 7.0463-7.0276 (m, 2H), 6.0285-5.8945 (m, 1H), 5.1759-5.118 (m, 2H), 3.9793 (s, 3H), 3.4550 (d, 2H, $J_{H13,H14}$ 6.45 Hz). ^{13}C NMR ($CDCl_3$ 75 MHz) δ 190.73, 158.18, 150.47, 148.07, 147.27, 143.55, 141.58, 137.69, 136.17, 130.28, 125.77, 123.78, 120.20, 118.59, 117.32, 116.93, 56.51, 39.81.

1.2.3. Synthesis of 3-(4-aminobenzoyl)-8-methoxy-6-(prop-2-en-1-yl)-2H-chromen-2-one (A5)

Derivative **A4** (1 eq) and tin chloride (II) dihydrate (5 eq) were added to ethanol (20 mL) and the mixture was stirred under reflux. After 2 h, TLC analysis (hexanes : ethyl acetate, 7:3, v/v) showed the reaction completion and the mixture was cooled to room temperature. Aqueous $NaHCO_3$ was then added to raise pH to eight and the mixture was extracted with ethyl acetate. The resulting organic phase was washed with brine, dried with anhydrous sodium sulfate, and the solvent evaporated in a rotary evaporator, leading to the pure product.

Dark Orange solid; yield 78%. M.p.: 259-261 °C. IR (ATR) 3490 and 3375 (ν N-H), 3047 (ν C-H ar), 2964 (ν C-H sp^2), 1704 (ν C=O ester), 1628 (ν C=O ketone). ^{1}H NMR ($CDCl_3$ 300 MHz) δ 7.8824 (s, 1H), 6.9754 (s, 1H), 6.9398 (s, 1H), 3.9728 (s, 3H), 3.4379 (d, 2H, $J_{H13,H14}$ 6.57 Hz), 6.0334-5.8985 (m, 1H), 5.1624-5.1042 (m, 2H), 7.7314 (d, 2H, $J_{H2',H3'}$ 8.61 Hz), 6.6214 (d, 2H $J_{H3',H2'}$ 8.61 Hz), 4.2652 (s, 2H).^{13}C NMR ($CDCl_3$ 75 MHz) δ189.56, 158.47, 152.26, 147.20, 144.14, 142.91, 137.05, 136.50, 132.69, 128.47, 126.34, 119.58, 118.93, 117.04, 115.62, 113.87, 56.47, 39.89.

1.2.4. Synthesis of 4-((4-(8-methoxy-6-(prop-2-en-1-yl)-2H-chromen-3-carbonyl)phenyl)amino)-4-oxobuta-noic acid (A6)

To a stirring solution of succinic anhydride (5 eq) in pyridine (5 mL), it was added the derivative **A5** (1 eq) and then the reaction mixture was heated at 90 °C for 24 h. When all the amine was consumed (TLC with hexanes : ethylacetate : methanol, 6:3.75:0.25, v/v/v), the reaction mixture was cooled to room temperature and the pH brought to 2 by addition of 1 M HCl. The formed solid was collected by vacuum filtration and purified by trituration with ethyl ether.

Light purple solid; yield 52%. M.p.: 172-174 °C. IR (ATR) 3296 (ν N-H amide), 3003 (ν C-H sp^2), 2970 (ν C-H ar), 1702 (ν C=O ester), 1660 (ν C=O ketone). ^{1}H NMR ($CDCl_3$ 300 MHz) δ 8.0316 (s, 1H), 7.9864-7.9575 (m, 2H), 7.4966-7.4678 (m, 2H), 7.0195-7.0137 (m, 1H), 6.9841 (s, 1H), 6.0335-5.8992 (m, 1H), 5.1752-5.1086 (m, 2H), 3.9805 (s, 3H), 3.4489 (d, 2H, $J_{H13,H14}$ 6.66), 2.9246 (s, 4H).^{13}C NMR ($CDCl_3$ 75 MHz) δ 190.82, 175.67, 158.02, 147.29, 146.26, 137.37, 136.61, 136.33, 135.95, 130.56, 126.84, 126.36, 119.93, 118.64, 117.23, 116.39, 56.52, 39.88, 28.57.

2. IR AND NMR SPECTRA

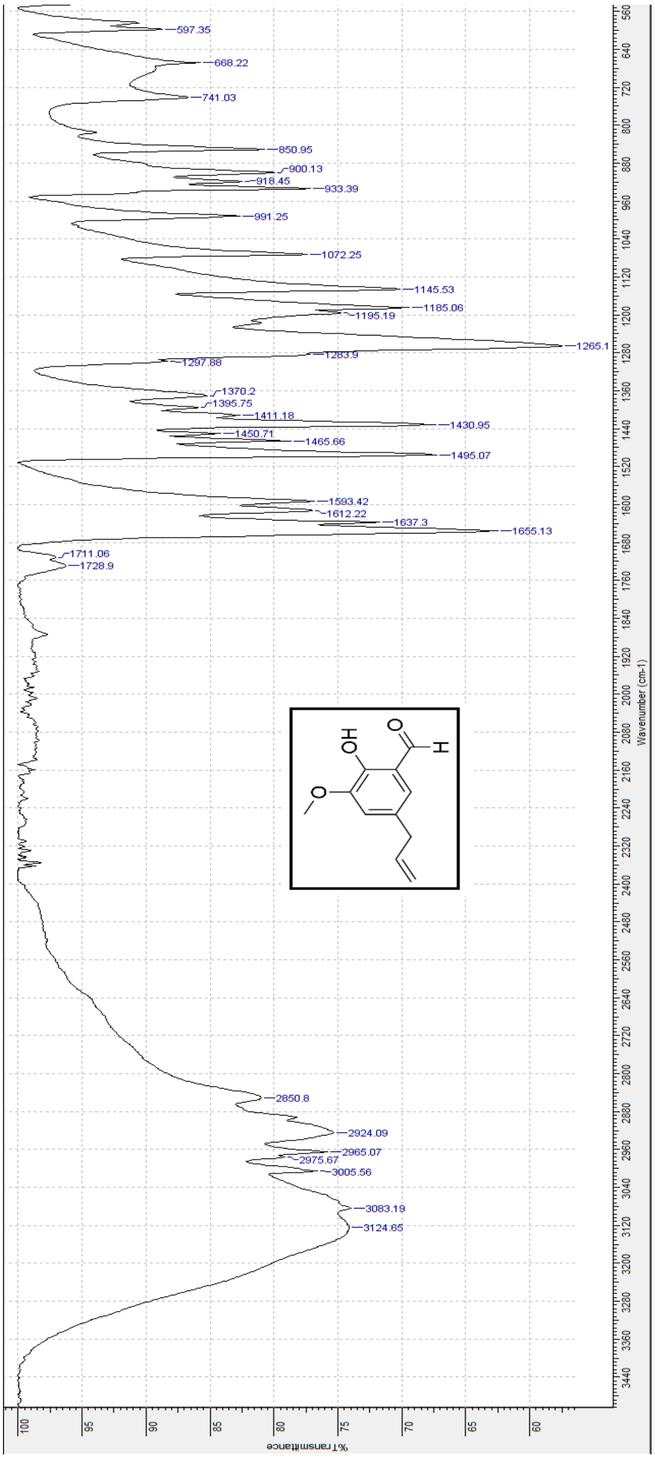

IR spectrum of formyleugenol.

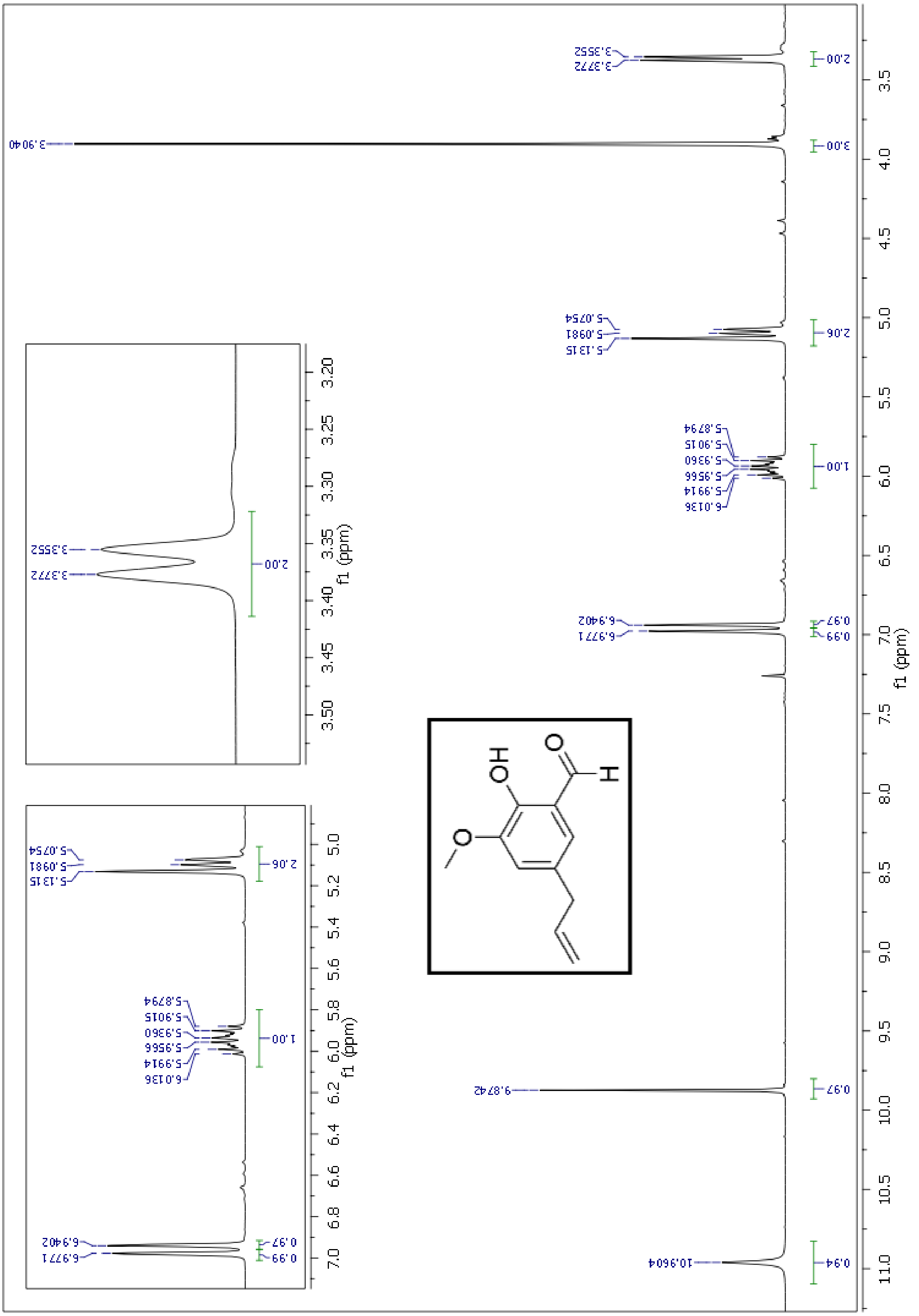

^{1}H NMR spectrum of formyleugenol ($CDCl_3$, 300 MHz).

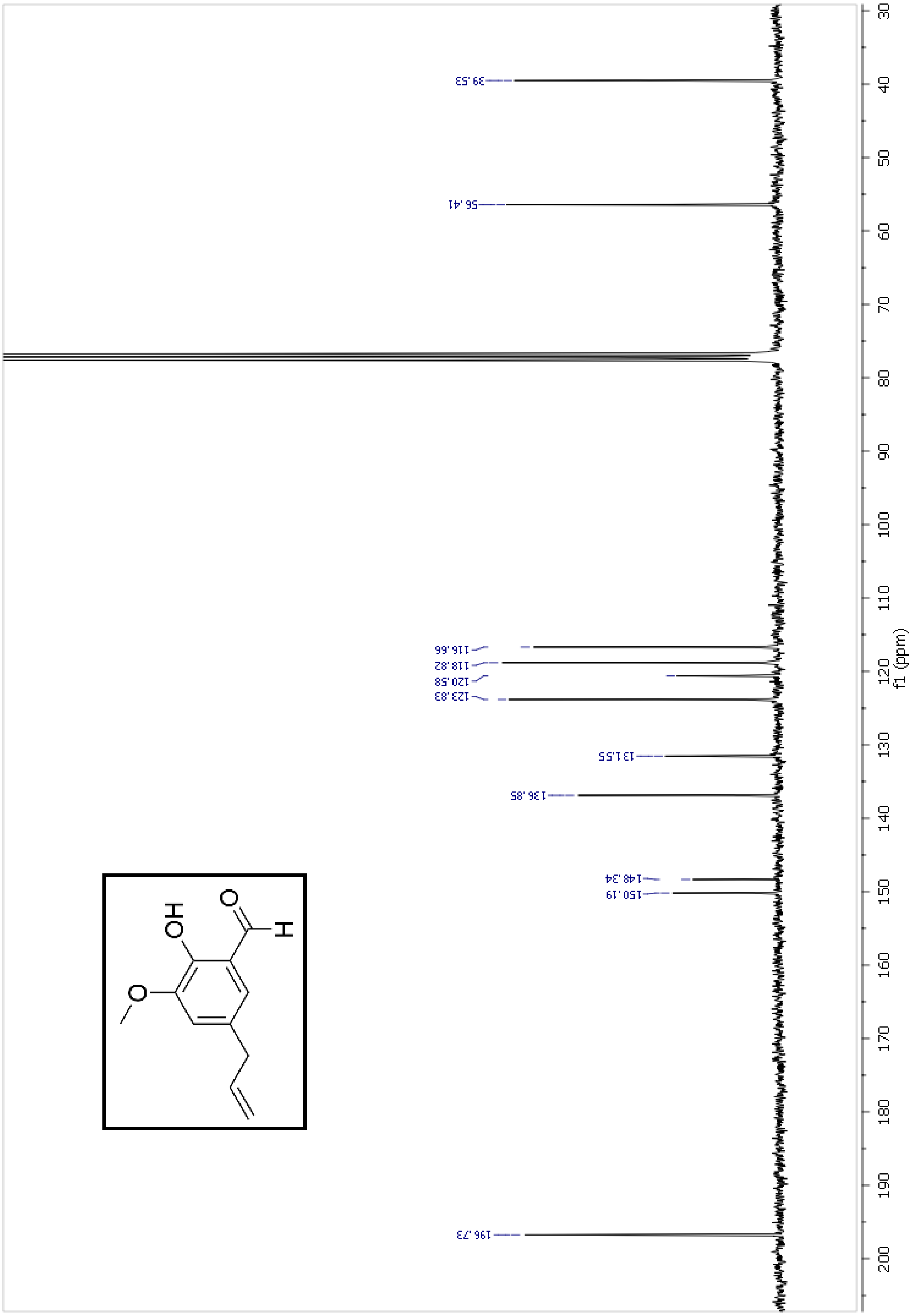

^{13}C NMR spectrum of formyleugenol ($CDCl_3$, 300 MHz).

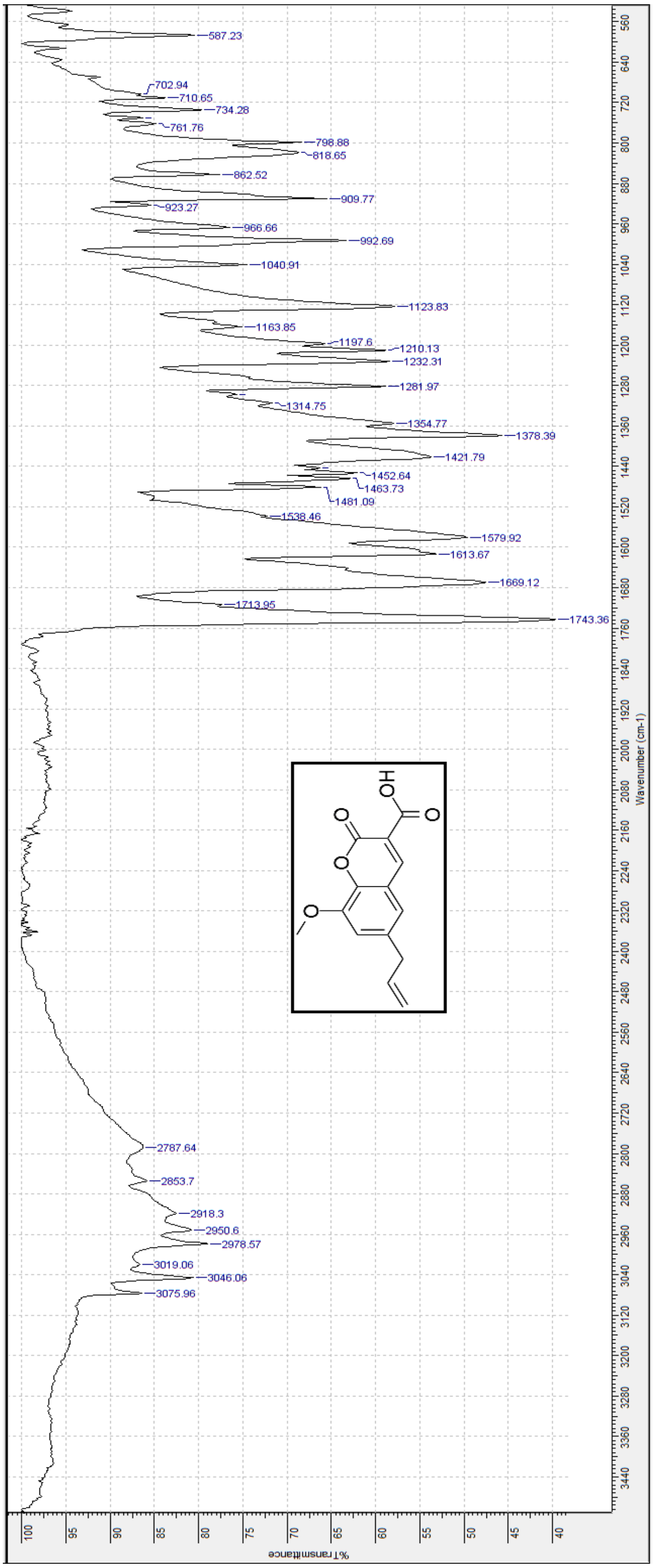

IR spectrum of coumarin A1.

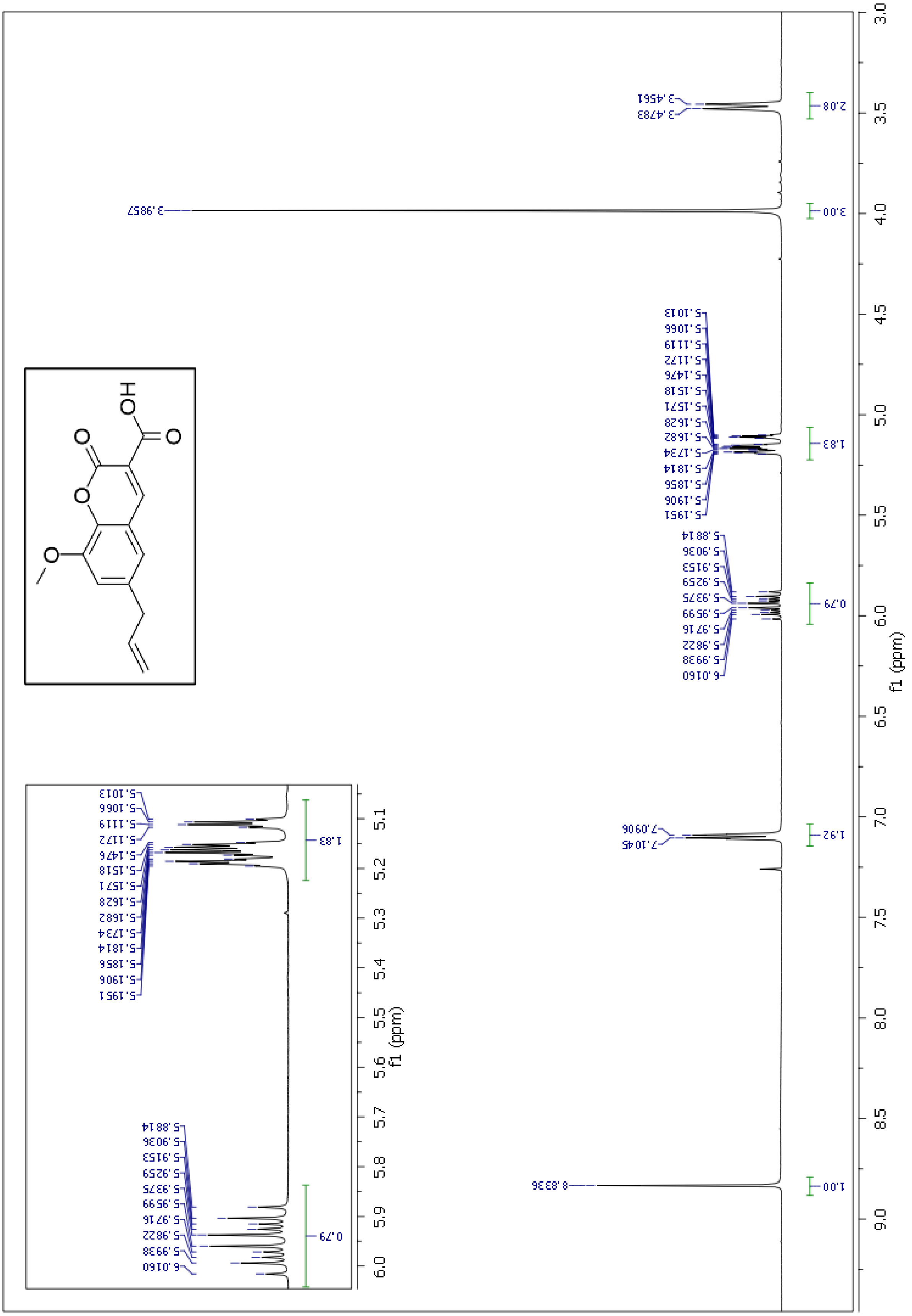

^{1}H NMR spectrum of coumarin A1 ($CDCl_3$, 300 MHz).

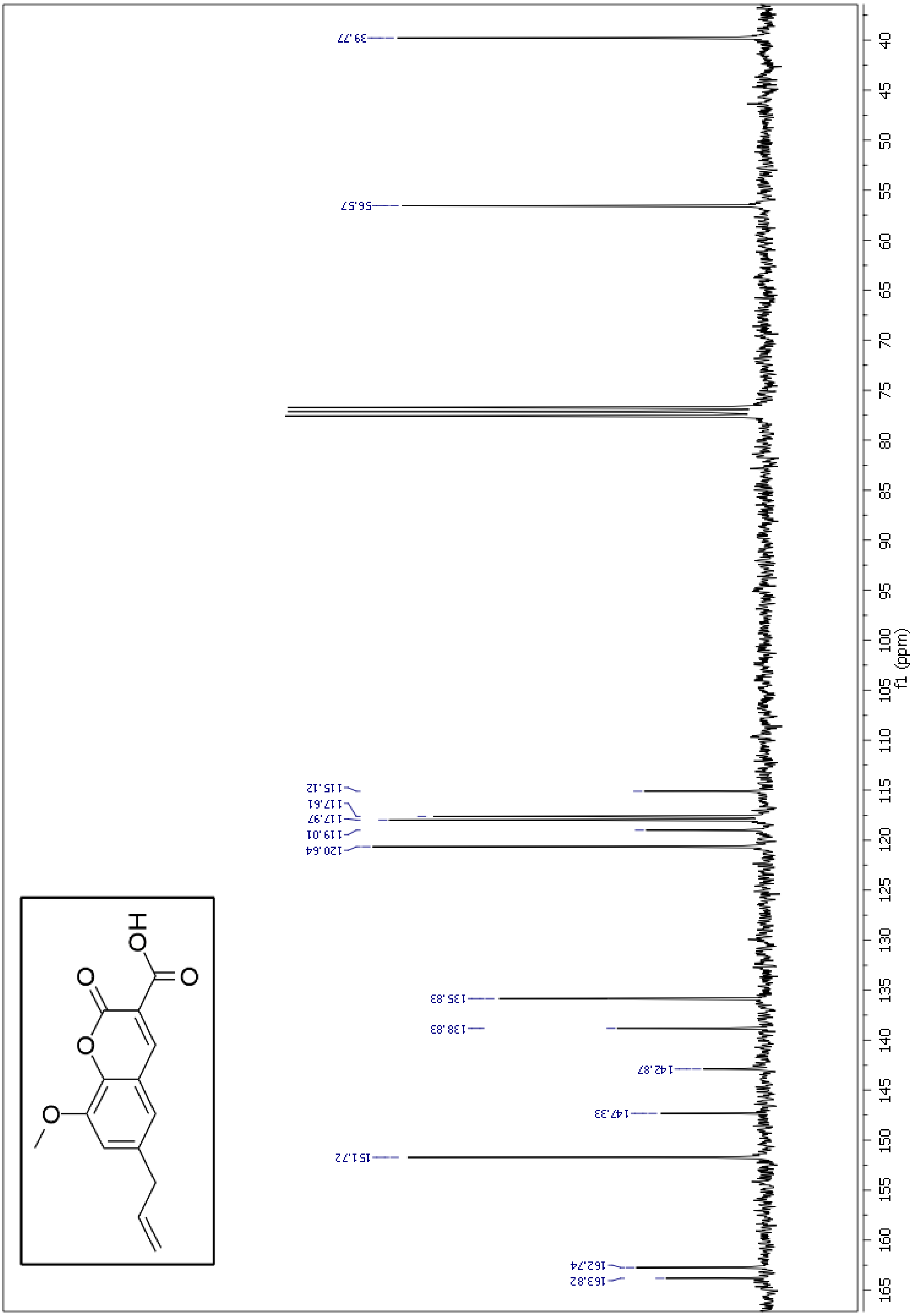

^{13}C NMR spectrum of coumarin A1 ($CDCl_3$, 300 MHz).

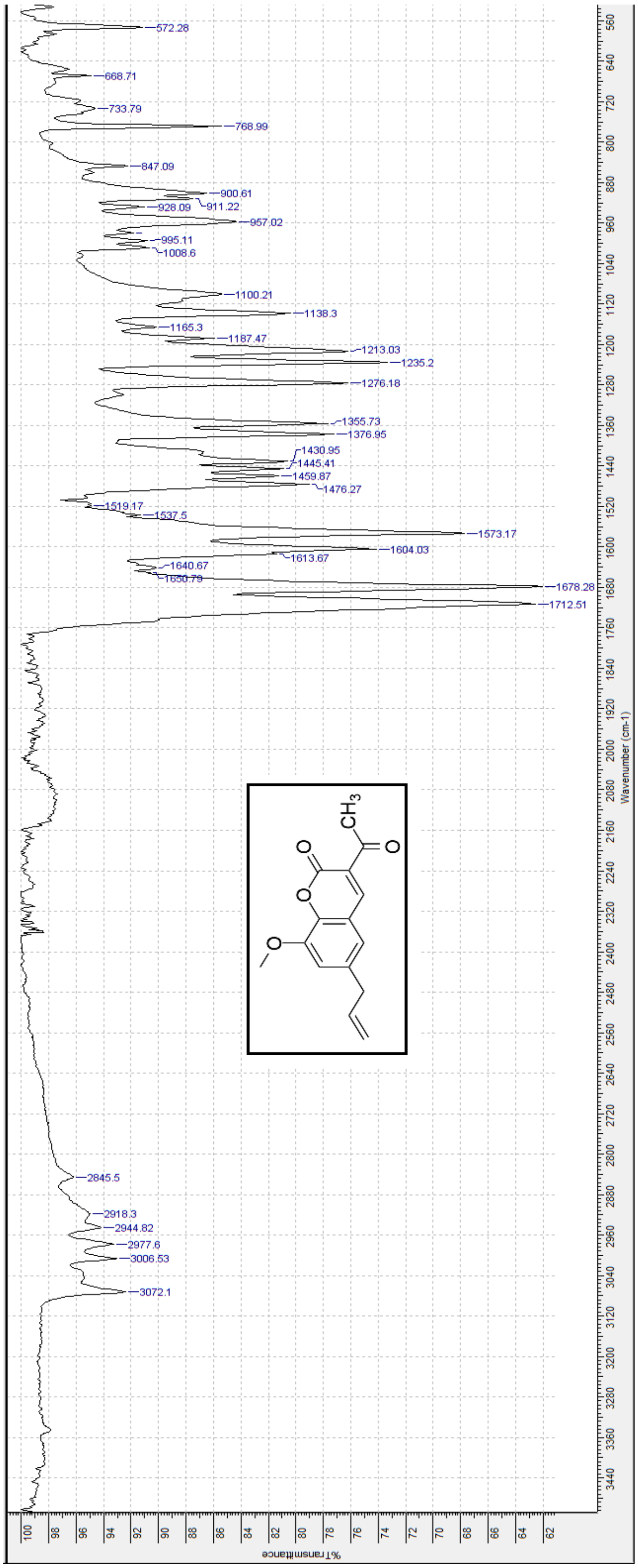

IR spectrum of coumarin A2.

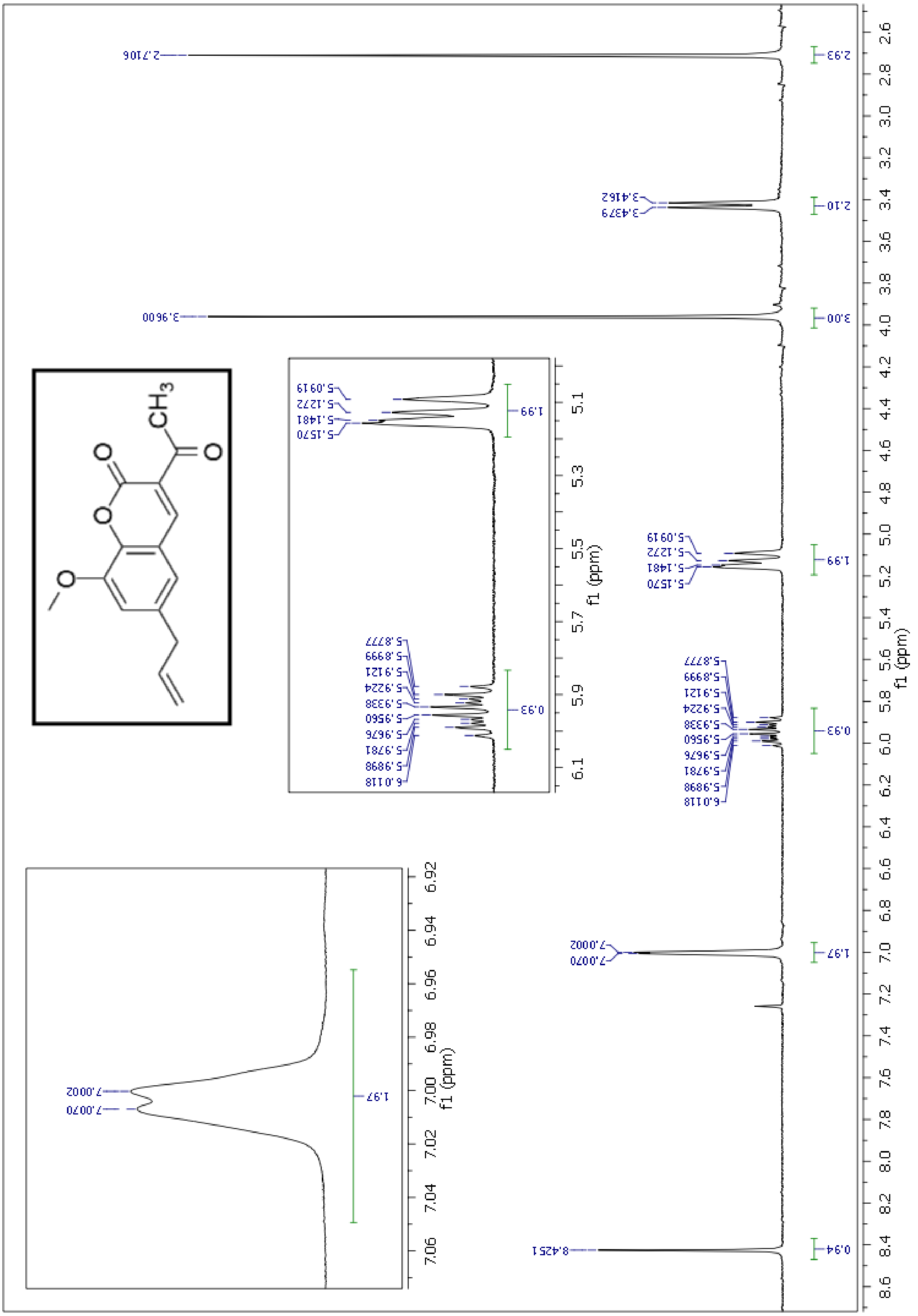

^{1}H NMR spectrum of coumarin A2 ($CDCl_3$, 300 MHz).

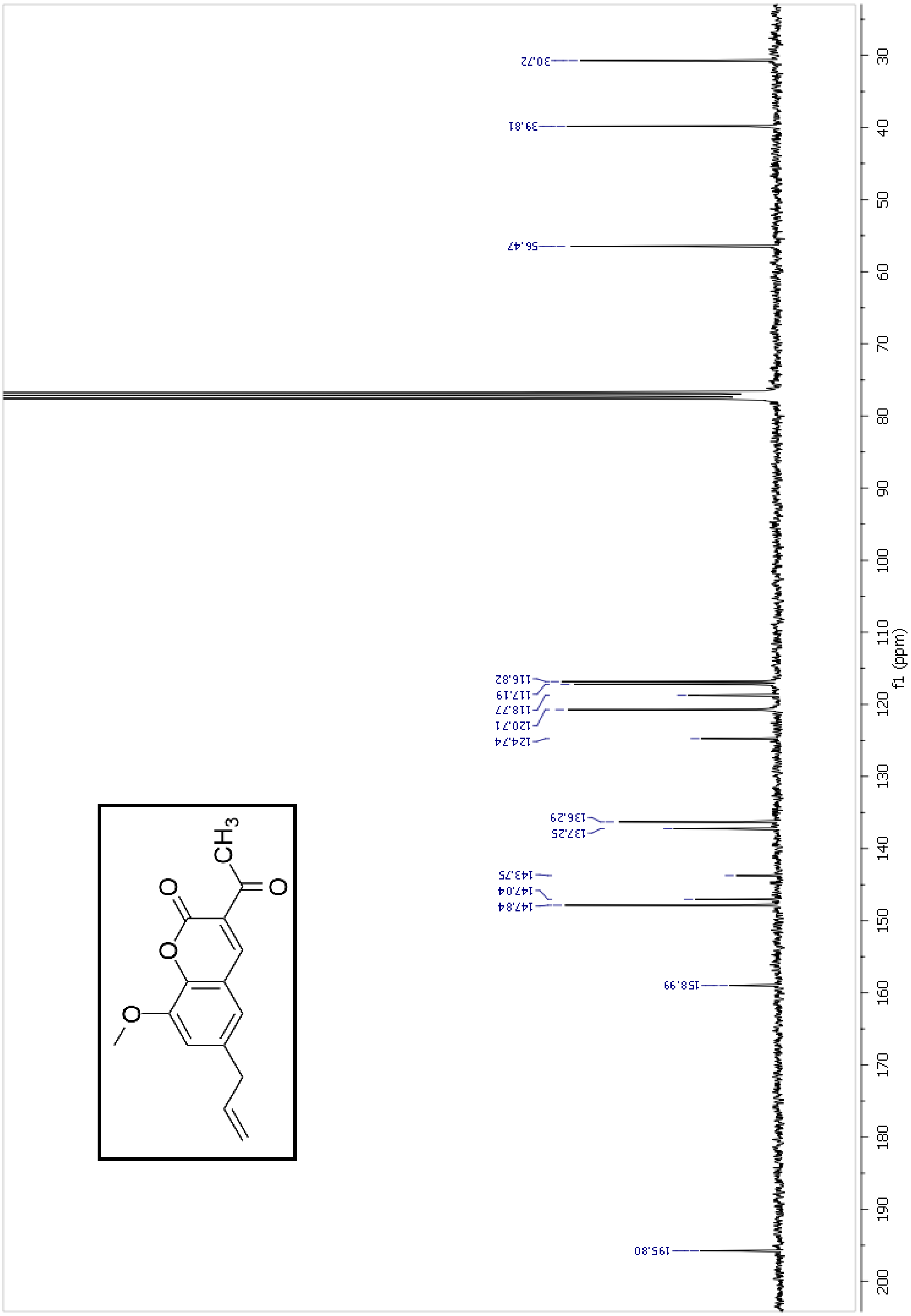

^{13}C NMR spectrum of coumarin A2 ($CDCl_3$, 300 MHz).

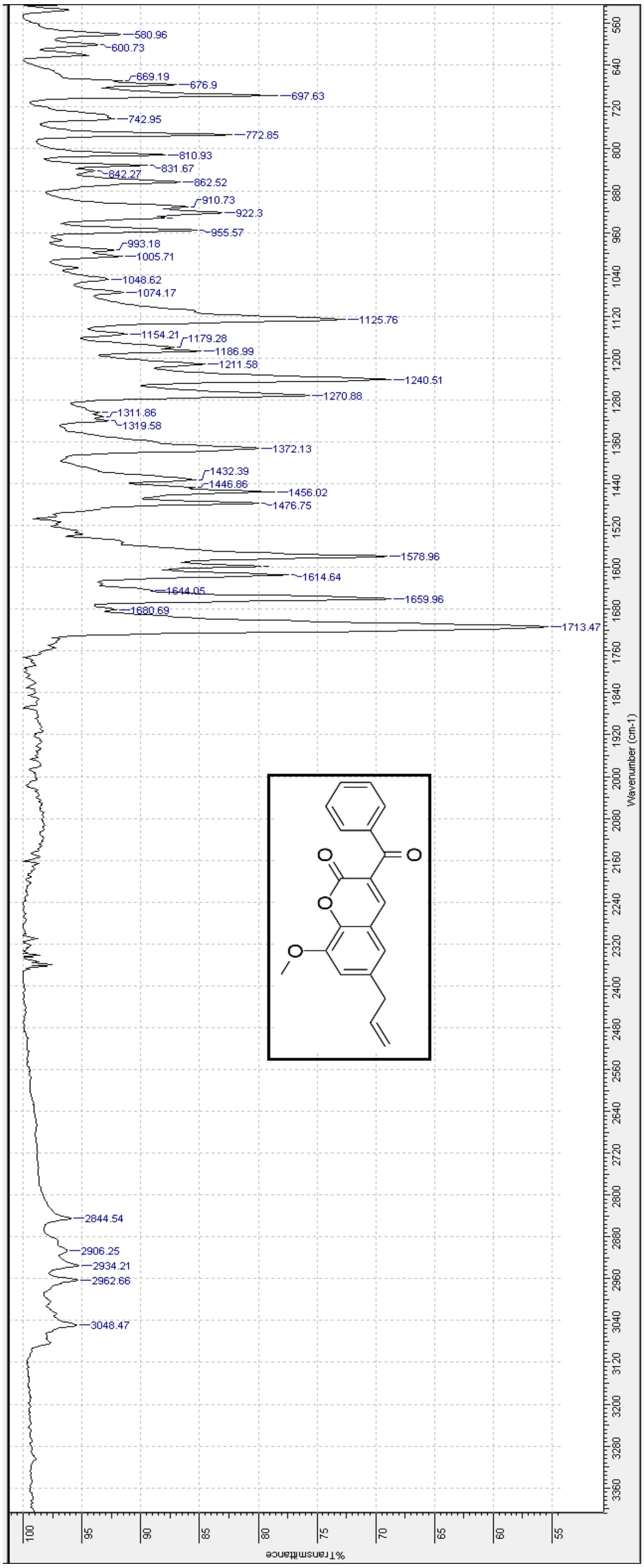

IR spectrum of coumarin A3.

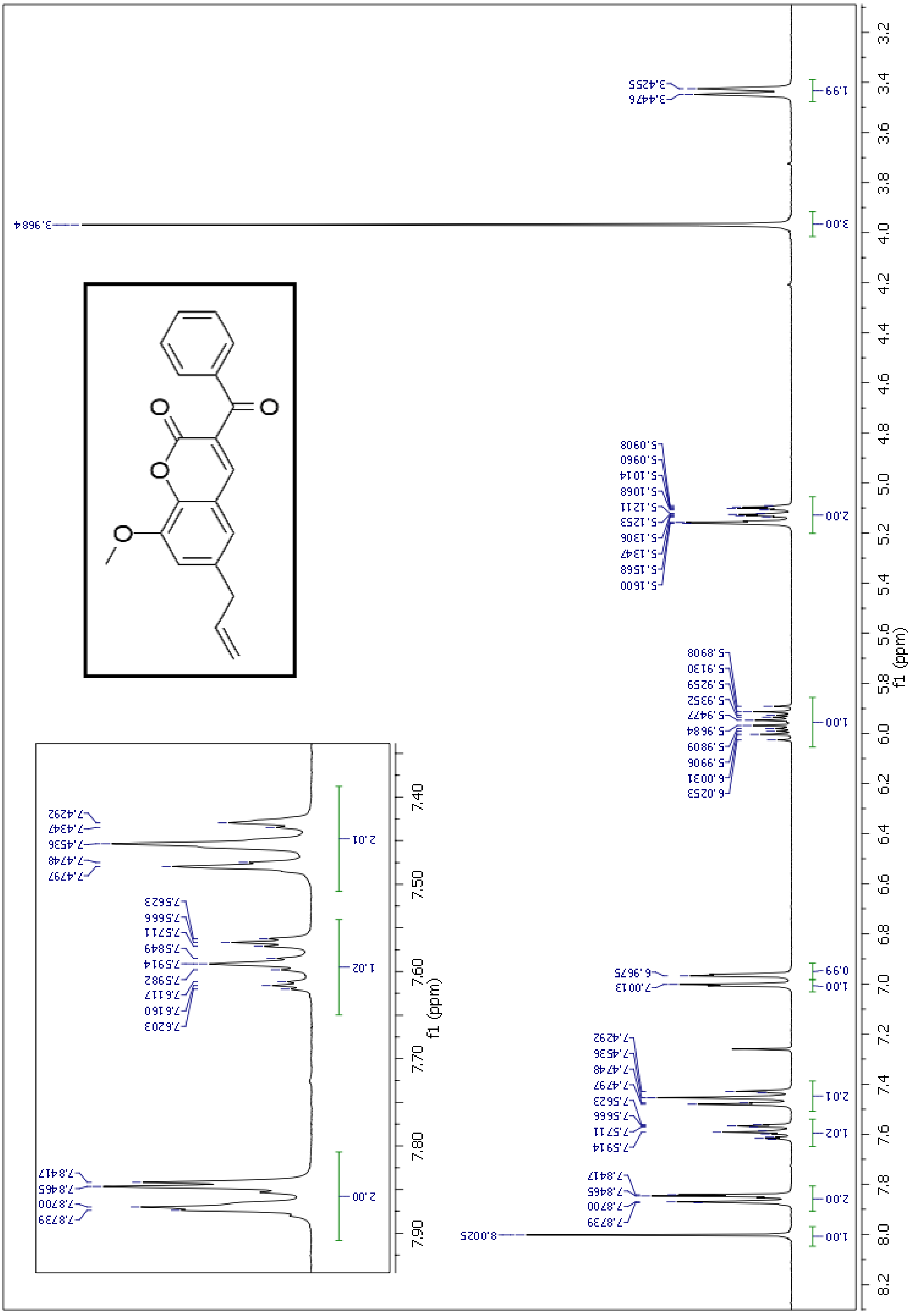

^{1}H NMR spectrum of coumarin A3 ($CDCl_3$, 300 MHz).

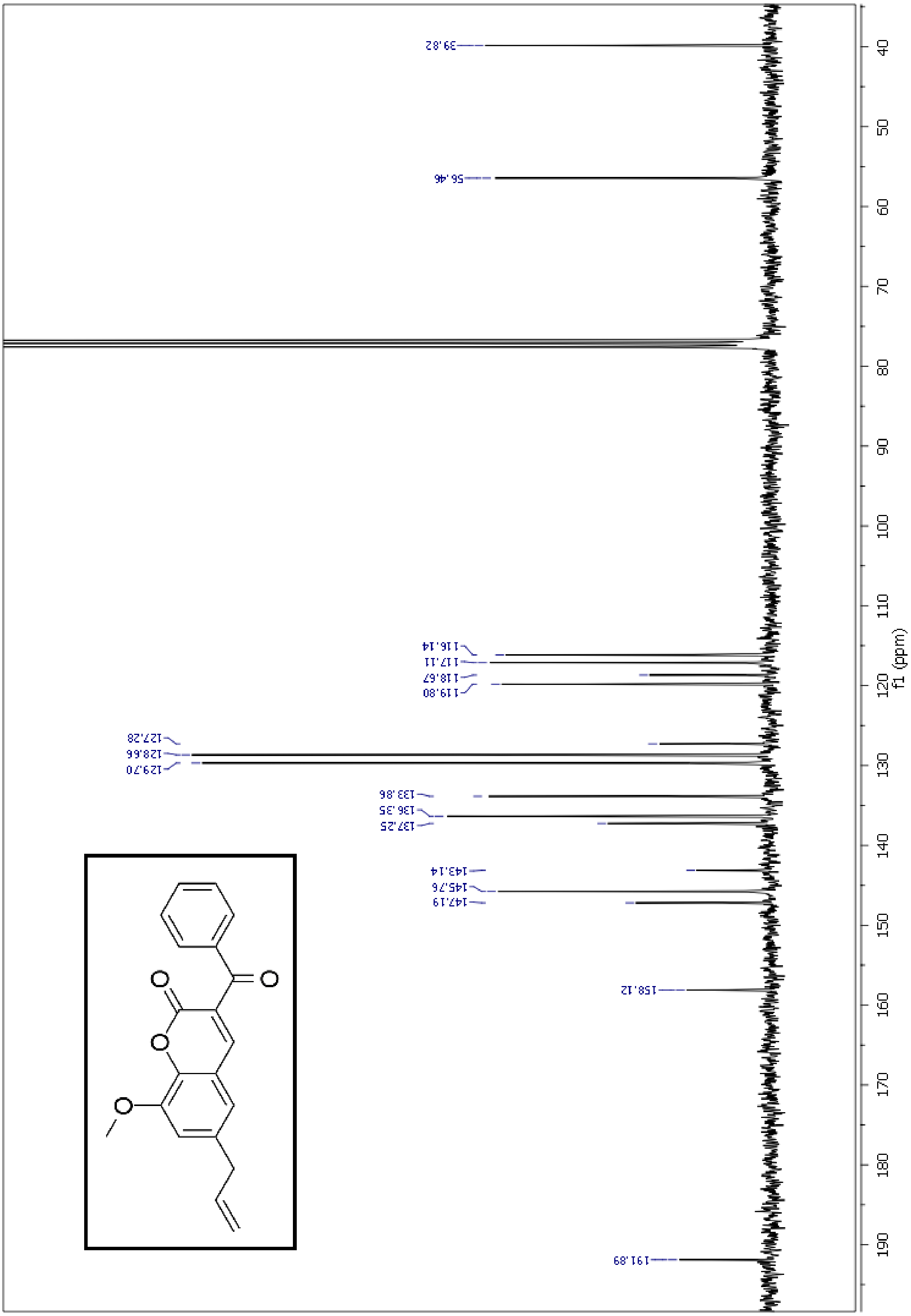

^{13}C NMR spectrum of coumarin A3 ($CDCl_3$, 300 MHz).

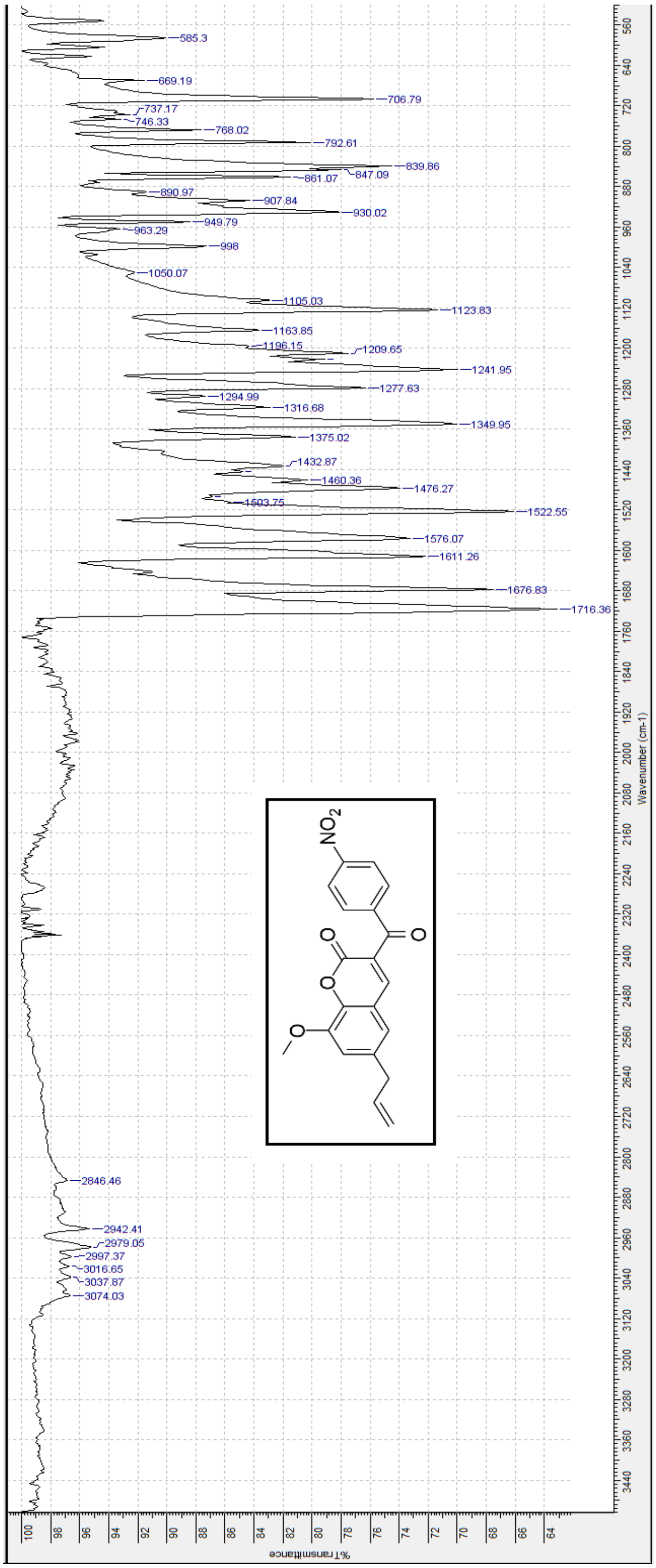

IR spectrum of coumarin A4.

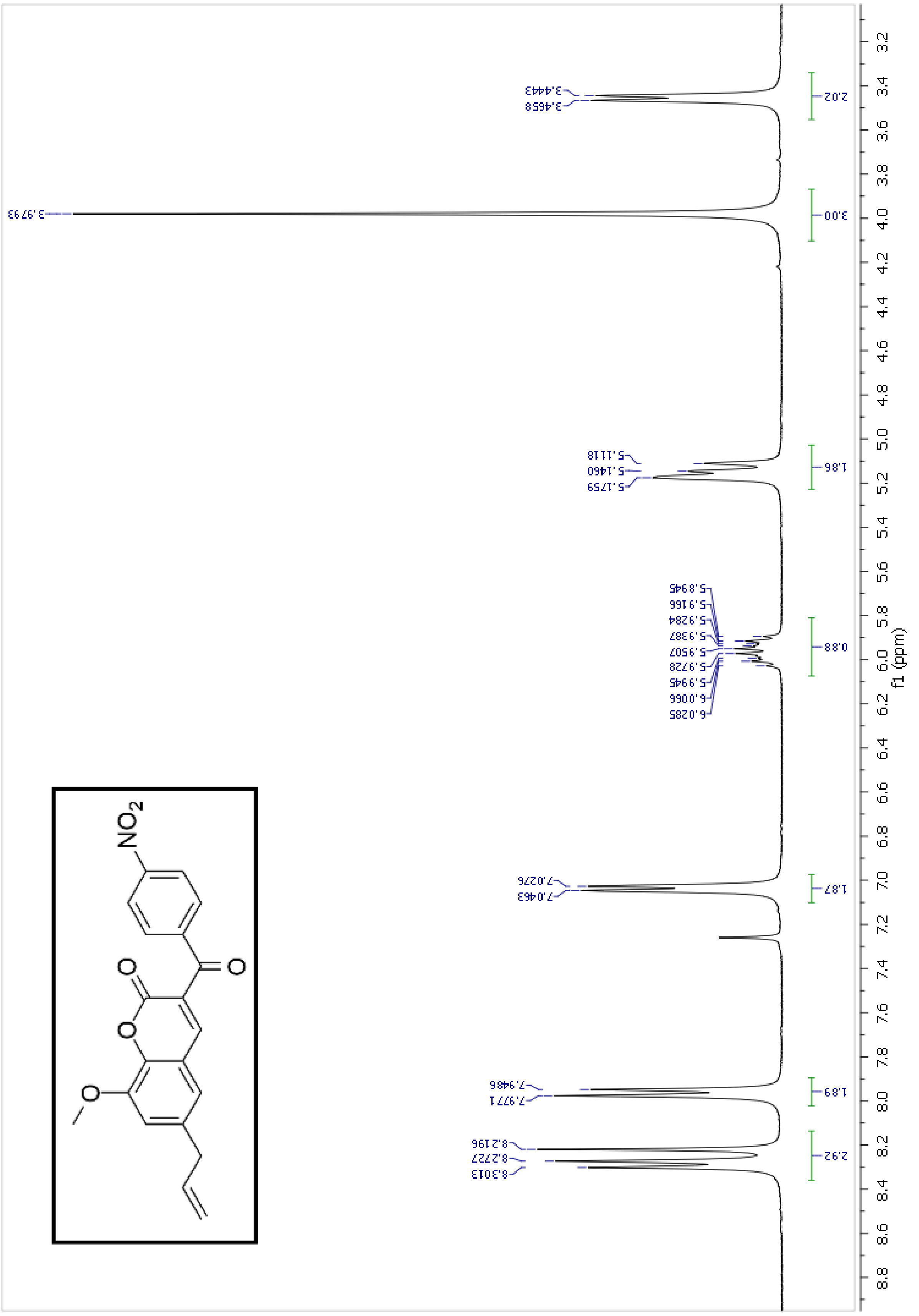

^{1}H NMR spectrum of coumarin A4 ($CDCl_3$, 300 MHz).

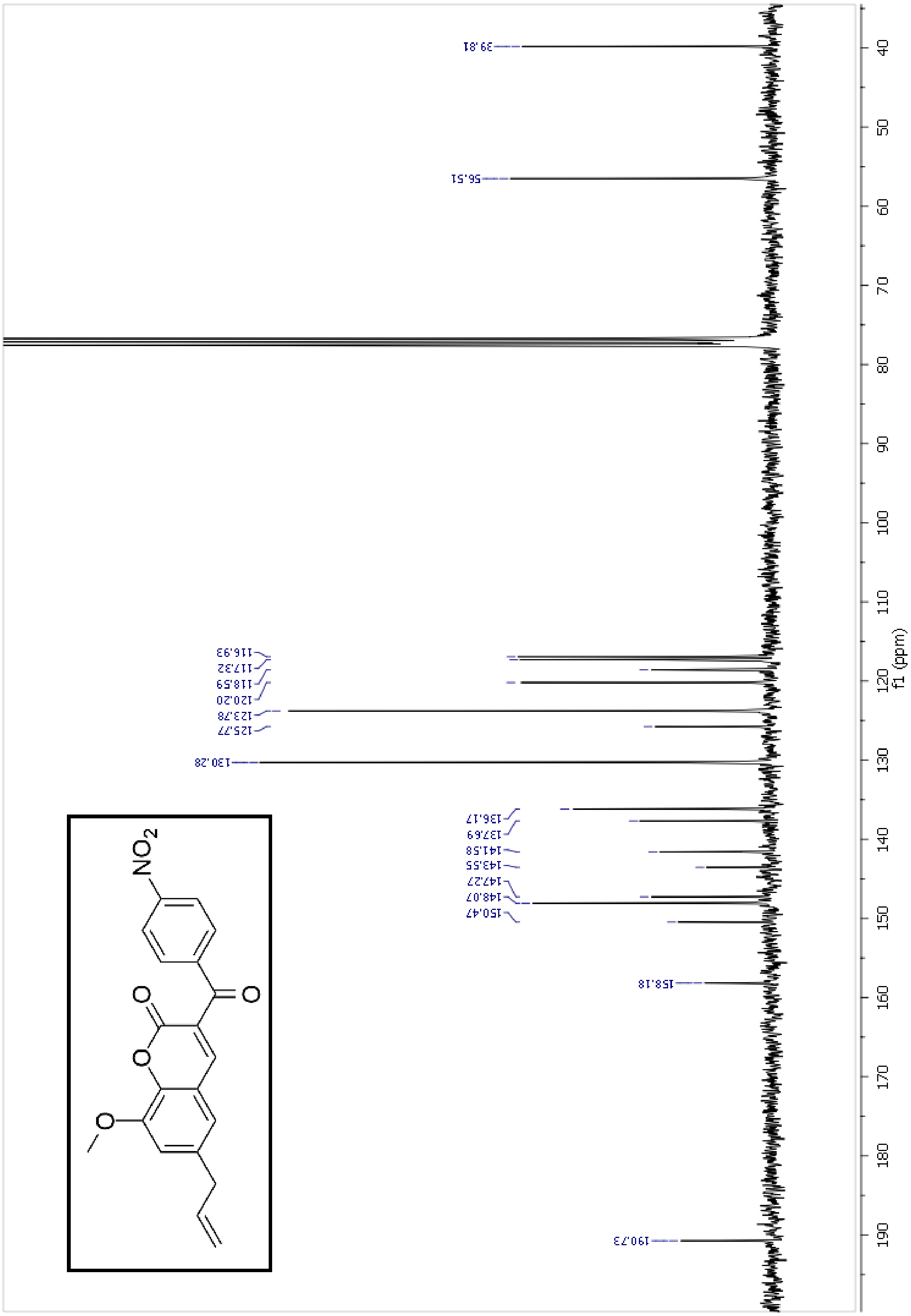

^{13}C NMR spectrum of coumarin A4 ($CDCl_3$, 300 MHz).

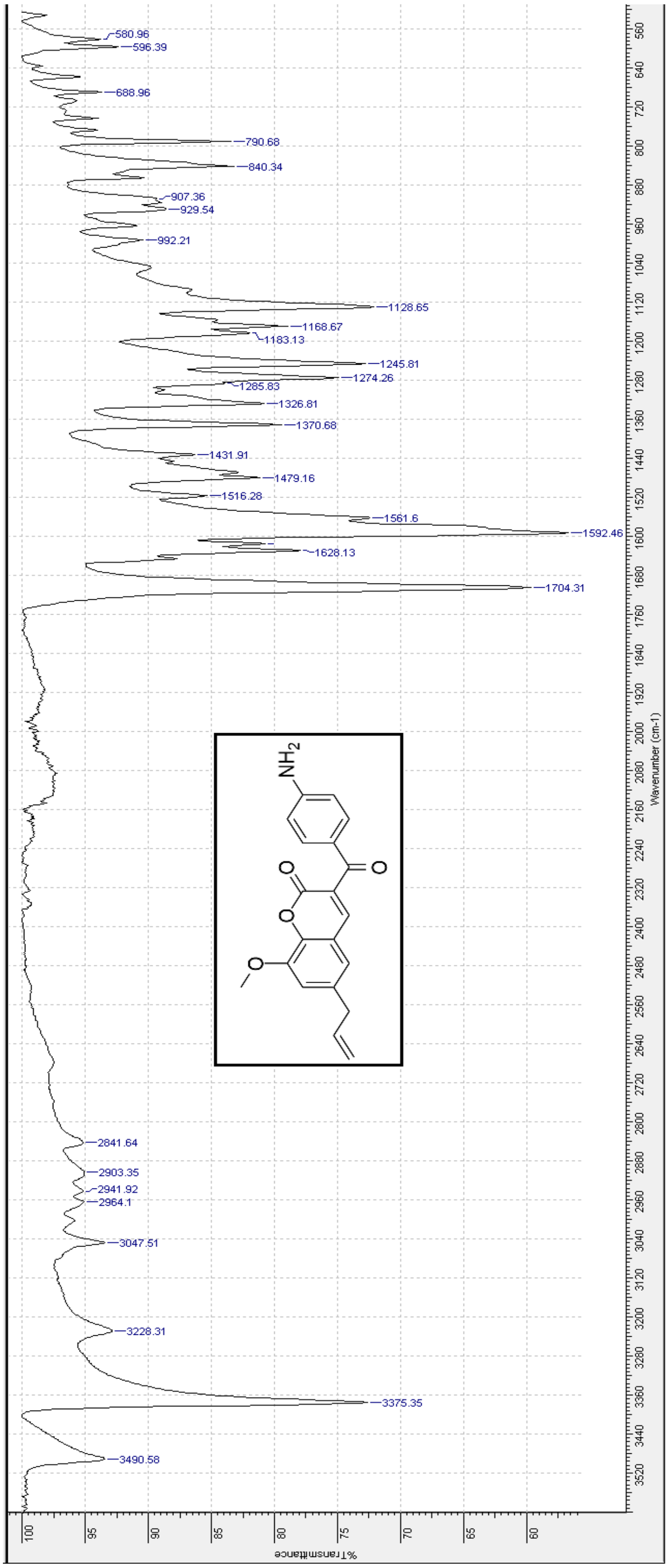

IR spectrum of coumarin A5.

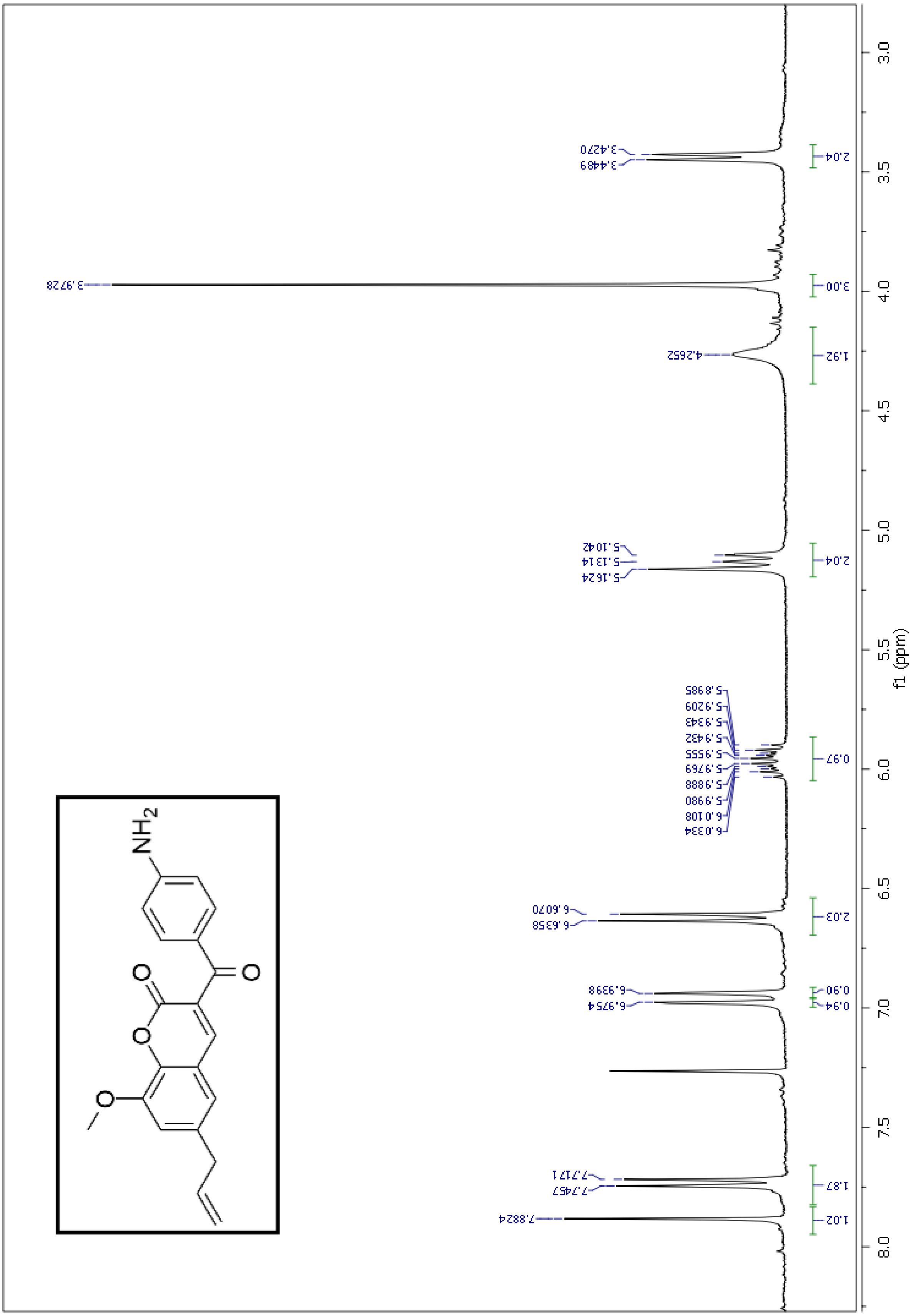

^{1}H NMR spectrum of coumarin A5 ($CDCl_3$, 300 MHz).

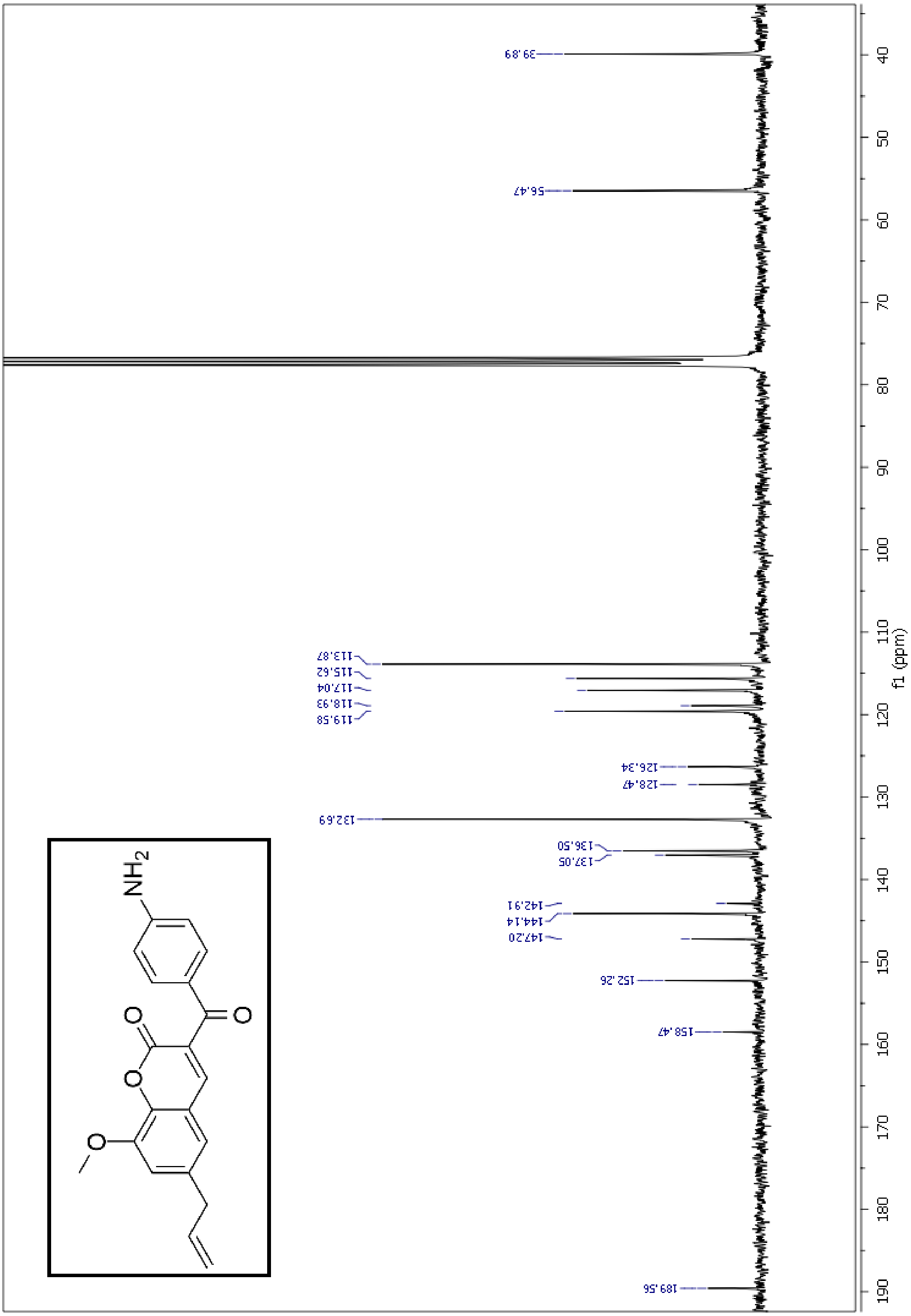

^{13}C NMR spectrum of coumarin A5 ($CDCl_3$, 300 MHz).

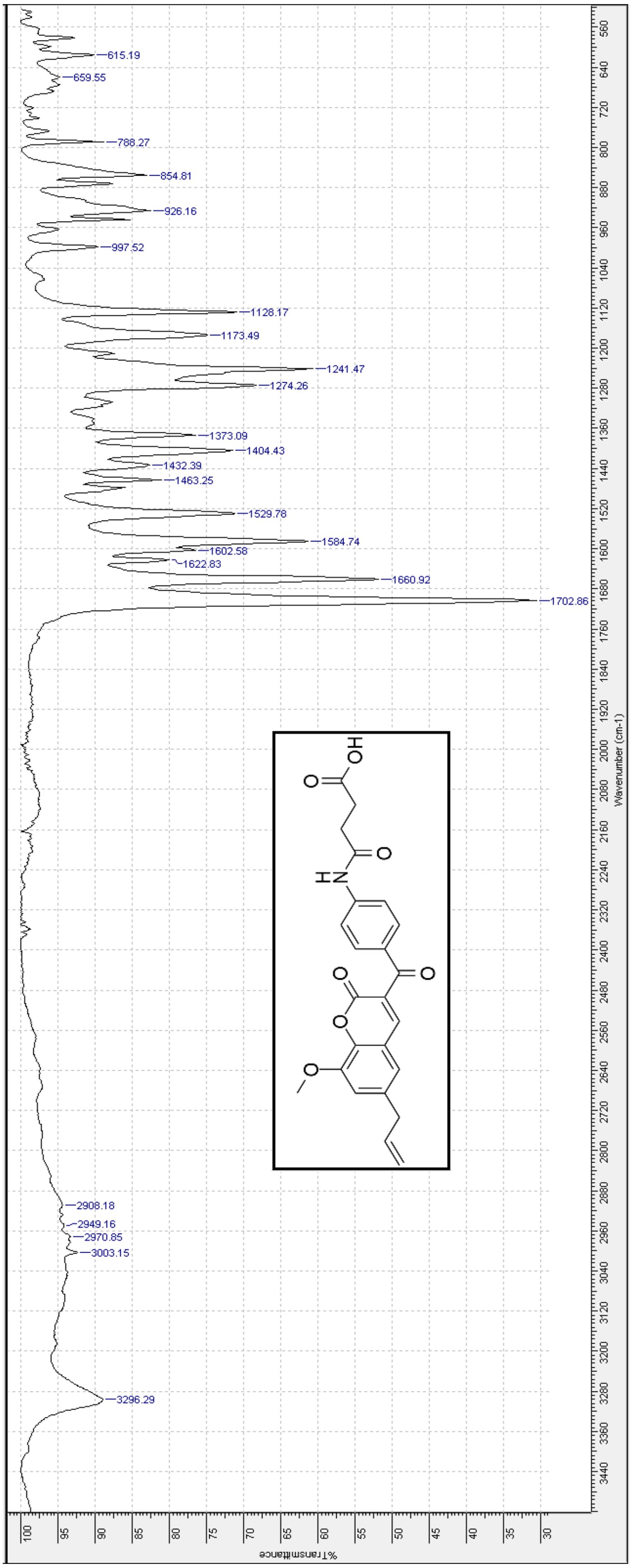

IR spectrum of coumarin A6.

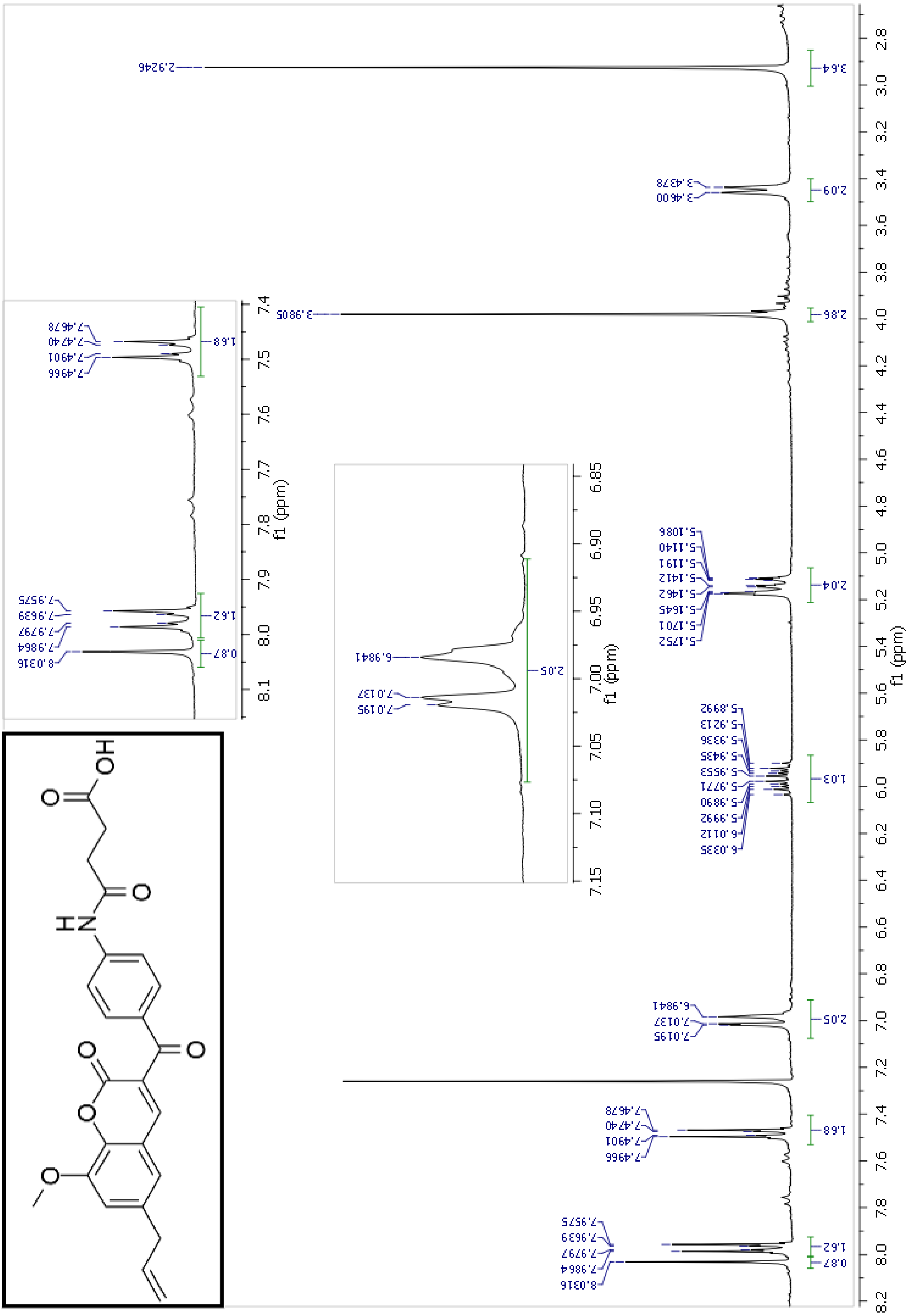

^{1}H NMR spectrum of coumarin A6 ($CDCl_3$, 300 MHz).

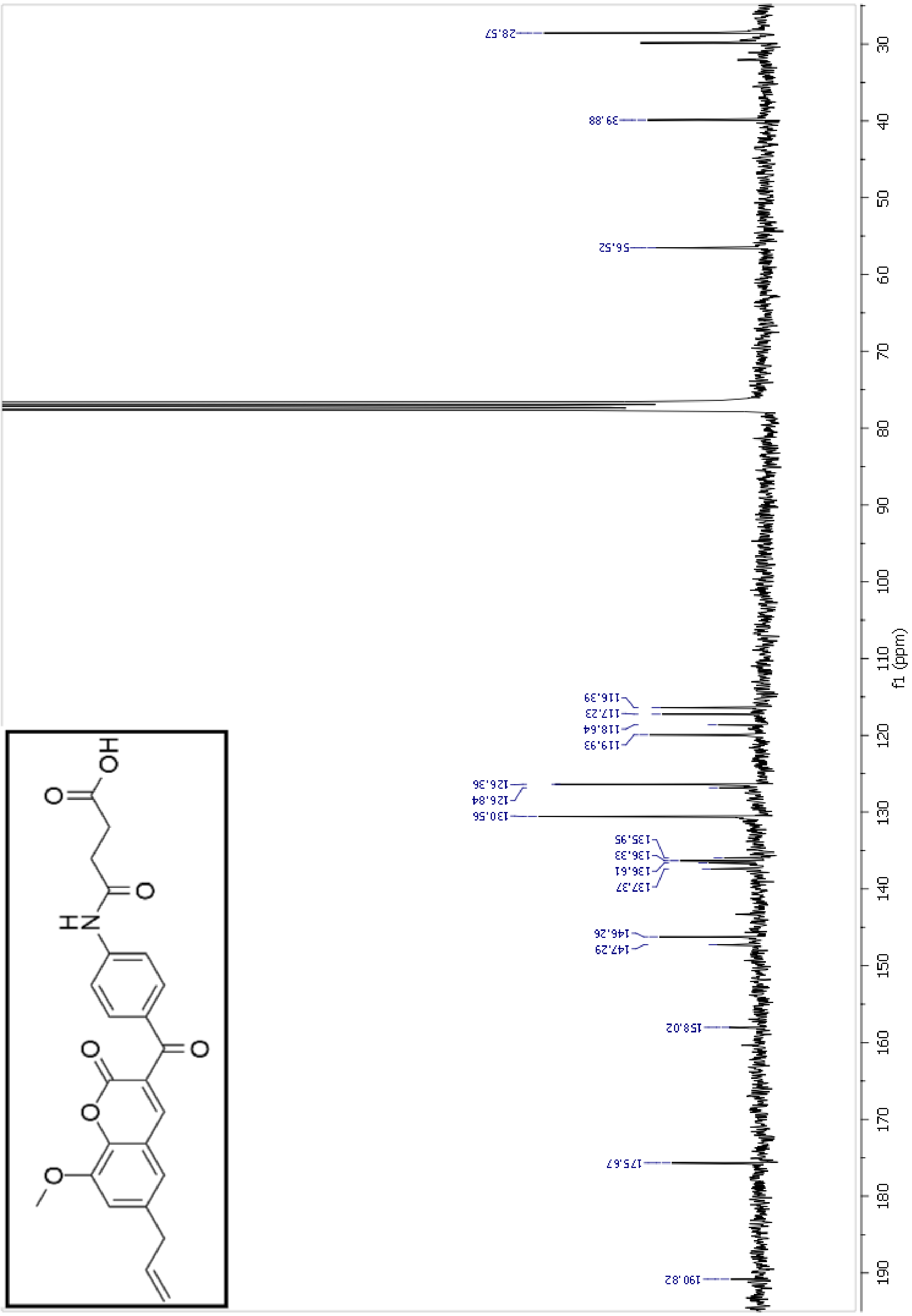

^{13}C NMR spectrum of coumarin A6 ($CDCl_3$, 300 MHz).

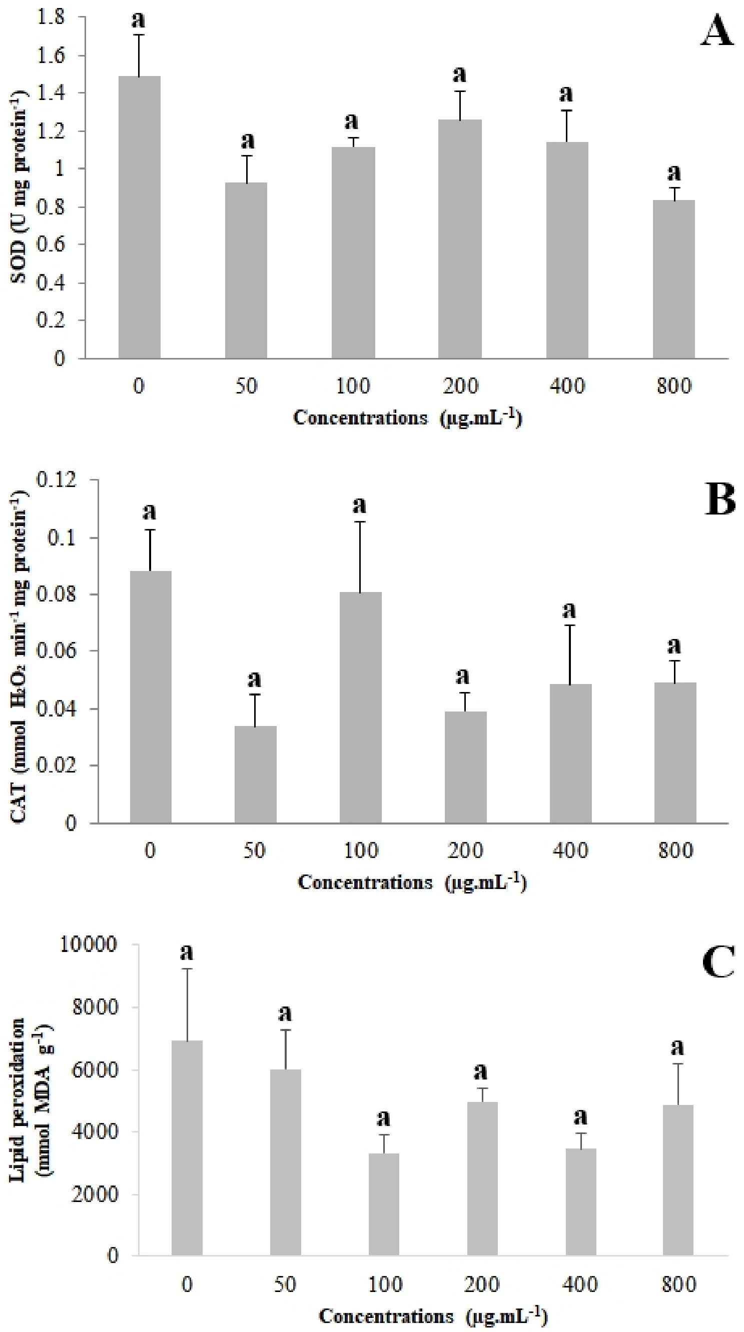

Figure S1: Antioxidant enzyme activity and lipid peroxidation. **(a)** Superoxide dismutase (SOD) activity in *Lactuca sativa* seedlings exposed to the different concentrations of coumarin A1. **(b)** Catalase (CAT) activity in *Lactuca sativa* seedlings exposed to the different concentrations of coumarin A1. **(c)** Quantification of lipid peroxidation in seedlings of *Lactuca sativa* exposed to the different concentrations of coumarin A1. Columns followed by the same letter do not differ statistically by the Scott-Knott test at 5% significance. Bar: standard error.
